# Off-label intrathecal use of gadobutrol: safety study and comparison of administration protocols

**DOI:** 10.1007/s00234-020-02519-4

**Published:** 2020-08-15

**Authors:** Merete Halvorsen, Camilla Sæthre Edeklev, Jorunn Fraser-Green, Grethe Løvland, Svein Are Sirirud Vatnehol, Øivind Gjertsen, Bård Nedregaard, Ruth Sletteberg, Geir Ringstad, Per Kristian Eide

**Affiliations:** 1 Department. of Neurosurgery, Oslo University Hospital - Rikshospitalet, Postboks 4950 Nydalen, 0424 Oslo, Norway; 2 The Interventional Centre, Oslo University Hospital - Rikshospitalet, Oslo, Norway; 3 Dept. of Radiology and Nuclear Medicine, Oslo University Hospital - Rikshospitalet, Oslo, Norway; 4 Faculty of Medicine, Institute of Clinical Medicine, University of Oslo, Oslo, Norway

**Keywords:** Glymphatic function, Magnetic resonance imaging, Contrast agents, Gadobutrol, Iodixanol, Safety

## Abstract

**Purpose:**

Magnetic resonance imaging (MRI) contrast agents have been used off-label for diagnosis of cerebrospinal fluid (CSF) leaks and lately also for assessment of the glymphatic system and meningeal lymphatic drainage. The purpose of this study was to further evaluate the short- and long-term safety profile of intrathecal MRI contrast agents.

**Methods:**

In this prospective study, we compared the safety profile of different administration protocols of intrathecal gadobutrol (Gadovist^TM^; 1.0 mmol/ml). Gadobutrol was administered intrathecal in a dose of 0.5 mmol, with or without iodixanol (Visipaque^TM^ 270 mg I/ml; 3 ml). In addition, a subgroup was given intrathecal gadobutrol in a dose of 0.25 mmol. Adverse events were assessed at 1 to 3 days, 4 weeks, and after 12 months.

**Results:**

Among the 149 patients, no serious adverse events were seen in patients without history of prior adverse events. The combination of gadobutrol with iodixanol did not increase the occurrence of non-serious adverse events after days 1–3. Intrathecal gadobutrol in a dose of 0.25 mmol caused less severity of nausea, as compared with the dose of 0.5 mmol. The clinical diagnosis was the major determinant for occurrence of non-serious adverse events after intrathecal gadobutrol.

**Conclusion:**

This prospective study showed that intrathecal administration of gadobutrol in a dose of 0.5 mmol is safe. Non-serious adverse events were to a lesser degree affected by the administration protocols, though preliminary data are given that side effects of intrathecal gadobutrol are dose-dependent.

## Introduction

Since the glia-lymphatic (or glymphatic) system was described in 2012 [1], it has attracted a lot of interest and scientific discussion by providing a new perspective on brain-wide clearance of toxic brain metabolites such as amyloid-β and tau protein and thereby playing a pivotal role in neurodegenerative disease [2]. Later, intrathecal contrast-enhanced magnetic resonance imaging (MRI) gave in vivo evidence for the existence of a brain-wide glymphatic system in humans [3, 4] and also demonstrated molecular efflux pathways from cerebrospinal fluid (CSF) to neck lymph nodes [5], the parasagittal dura [6], and choroid plexus [7].

Several concerns exist about the off-label intrathecal use of MRI contrast agents, primarily due to potential neurotoxic effects [8–10], and report on retention of linear gadolinium chelates within the human brain extra-vascular space after repeated intravenous administrations [11–13]. A potential mechanism for the findings of gadolinium outside the blood-brain barrier was given by recent observations of MRI contrast agents leaking from blood to CSF [14, 15], even in individuals without blood-brain barrier dysfunction [15, 16], mainly through the choroid plexus [17], but also via the ciliary body [16, 18]. In principle, an intravenous dosage of contrast agent to venous blood therefore also represents a small dosage of contrast agent to CSF; one MRI study indicated that CSF concentration of contrast agent is one-fifth of the intravenous concentration after intravenous administration [16].

We recently reported the safety profile of intrathecal gadobutrol in 100 subjects [19]. The purpose of the present study was to expand the analysis to include additional 49 patients in order to compare different administration protocols of intrathecal gadobutrol, i.e., 0.5 mmol gadobutrol (0.5 ml of Gadovist^TM^, 1.0 mmol/ml), with or without 3 ml iodixanol (Visipaque^TM^, 270 mg I/ml). In addition, we performed MRI using 0.25 mmol gadobutrol. Adverse events, categorized as serious or non-serious, were assessed days 1–3 and after 4 weeks and 12 months.

## Materials and methods

### Patient material and study design

The study design was prospective and carried out during the period from September 2015 to December 2019. We included consecutively patients admitted to the Department of neurosurgery, Oslo University Hospital - Rikshospitalet, for work-up of various CSF circulation disorders (Table 1). Exclusion criteria were as follows: a history of hypersensitivity reactions to imaging contrast agents or a history of severe allergy reactions in general or evidence of renal dysfunction. Pregnant or breastfeeding women and individuals < 18 or > 80 years were not included.

**Table 1:** Patient material and different protocols

	Patient material	Protocol			Statistics	
	Total	#1	#2	#3	Protocol #1 vs. #2	Protocol #2 vs. #3
*N*	149	115	29	5		
Age (years)	48.6 ± 17.9	49.0 ± 18.7	46.9 ± 15.4	48.8 ± 15.2	ns	ns
Gender (female/male)	92 (62%)/57 (38%)	69 (60%)/46 (40%)	20 (69%)/9 (31%)	3 (60%)/2 (40%)	ns	ns
BMI (kg/m^2^)	27.5 ± 5.1	27.3 ± 5.1	28.1 ± 4.6	29.4 ± 7.9	ns	ns
Tentative diagnosis						
Idiopathic normal pressure hydrocephalus	38 (26%)	36 (31%)	1 (3%)	1 (20%)	*P* = 0.002	ns
Spontaneous intracranial hypotension	22 (15%)	15 (13%)	6 (21%)	1 (20%)	ns	ns
Arachnoid cyst	27 (18%)	19 (17%)	7 (24%)	1 (20%)	ns	ns
Pineal cyst	26 (17%)	23 (20%)	2 (7%)	1 (20%)	ns	ns
Idiopathic intracranial hypertension	22 (15%)	11 (10%)	10 (35%)	1 (20%)	*P* = 0.001	ns
Communicating hydrocephalus	11 (7%)	8 (7%)	3 (10%)	0	ns	ns
Non-communicating hydrocephalus	3 (2%)	3 (3%)	0	0	ns	-

Data given as numbers (percentage in parenthesis) and mean ± standard deviation. Significance determined by the Pearson chi-square test. *ns* non-significant

This research study was approved by the Institutional Review Board (2015/1868), Regional Ethics Committee (2015/96) and the National Medicines Agency (15/04932-7). Participants were included following written and oral informed consent.

### Protocols for intrathecal gadobutrol

Intrathecal contrast administration was performed by interventional neuroradiologists using x-ray guided lumbar puncture. Access to the spinal canal was typically obtained at level L2/L3 or L3/L4 and in some cases at level L4/5. Correct placement of a spinal syringe tip was secured by CSF backflow from the puncture needle (22G × 3½″) and the free distribution of radiopaque contrast agent in CSF when this was used. In this study, three different protocols were used:

#### Protocol #1

Intrathecal injection of 0.5 mmol (0.5 ml of 1.0 mmol/ml gadobutrol; Gadovist^TM^, Bayer Pharma AG, Berlin, Germany) in combination with 2–3 ml of 270 mg I/ml iodixanol (Visipaque^TM^, GE Healthcare, USA).

#### Protocol #2

Intrathecal injection of 0.5 mmol (0.5 ml of 1.0 mmol/ml gadobutrol; Gadovist^TM^, Bayer Pharma AG, Berlin, Germany) alone.

#### Protocol #3

Intrathecal injection of 0.25 mmol (0.25 ml of 1.0 mmol/ml gadobutrol; Gadovist^TM^, Bayer Pharma AG, Berlin, Germany) alone.

### MRI acquisitions

As previously described [20], the MRI protocol included acquisition of sagittal 3D T1-weighted spoiled gradient echo volume scans using a

3 T Philips Ingenia^TM^ MRI scanner (Philips Medical systems, Best, The Netherlands), with equal imaging sequence parameters at all time points. The following main imaging parameters were used: repetition time = “shortest” (typically 5.1 ms), echo time = “shortest” (typically 2.3 ms), flip angle = 8 degrees, field of view = 256 × 256 cm, and matrix = 256 × 256 pixels (reconstructed 512 × 512); 184 over-contiguous slices with 1 mm thickness automatically reconstructed to 368 slices and a thickness of 0.5 mm were sampled. Each image acquisition lasted about 6.5 min.

Consecutive and identical MRI acquisitions covering the cranial compartment and upper neck region were repeated at regular intervals, to verify and assess time of gadobutrol entry to cranial CSF spaces. The patients were transferred between the MRI suite and the ward department and between the bed and the MRI table by the hospital staff to keep the patient in the supine position. The participants were instructed to remain supine in bed the first hours after intrathecal gadobutrol but could move freely from afternoon. The MRI protocol included MRI acquisitions after 0–20 min, 20–40 min, 40–60 min, 1–2 h, 4–6 h, 6–9 h, 24 h, and 48 h.

The first appearance of MRI contrast agent in the subarachnoid space at level of the foramen magnum was visually assessed by an experienced neuroradiologist (G.R.) on the sagittal T1-weighted volume scans in the hospital PACS (Sectra IDS7®, Sectra, Sweden).

For quantitative assessment how different doses (0.5 versus 0.25 mmol) of intrathecal gadobutrol changed the intensity of the T1 signal, a region of interest was placed within the cisterna magna, with assessment of mean T1 signal intensity (in signal units) from the image gray scale. The signal unit was normalized against a reference region of interest by dividing measured T1 intensity from CSF with the value of the reference region of interest to correct for any baseline changes of image gray scale due to image scaling. The reference region of interest was placed within the posterior part of the superior sagittal sinus in axially reconstructed images from the same T1 volume scan.

### Assessment of serious and non-serious adverse events

The adverse events were categorized as serious or non-serious:

- Serious adverse events were any untoward medical occurrence resulting in death, being immediately life-threatening, requiring in-patient hospitalization or prolongation of existing hospitalization, resulting in persistent or significant disability or incapacity, or being an important medical event that may jeopardize the subject or may require medical intervention to prevent one of the mentioned outcomes.
- Non-serious adverse events were defined as any untoward medical occurrence in study participants, including any unfavorable and unintended sign, symptom, or temporary disease, whether or not related to the intrathecal gadobutrol administration. Participants were specifically asked for presence of defined symptoms chosen according to side effects most commonly observed after intrathecal iohexanol: headache (mild/moderate/severe), nausea (mild/moderate/severe), dizziness (mild/moderate/severe), itch, warmth feeling, paresthesia, visual symptoms, cognitive difficulties, muscular spasms, discomfort at injection site, and tremor. In addition, other complaints than those specifically questioned for were listed, independent of possible cause.

The adverse events were recorded systematically by study nurses not otherwise involved in management of patients at 1–3 days after intrathecal contrast agent administration and at 4 weeks. Patients who still reported symptoms at 4 weeks were phoned by the study nurses at a later occasion ranging from a few months to up to 12 months for a final assessment. Report of symptoms at any other contact after 4 weeks was registered and categorized as “Late.”

### Statistics

The statistical analyses were performed using the SPSS software version 25 (IBM Corporation, Armonk, NY). Significant differences between categorical data were determined using the Pearson chi-square test. Differences between groups were determined using one-way ANOVA. Significance was accepted at the 0.05 level.

## Results

### Patients

Table 1 presents demographic information and clinical indication for intrathecal gadobutrol in the 149 patients included in this study. The type of clinical indication differed between the different protocols.

### Verified intracranial distribution of gadobutrol

**Fig. 1:**
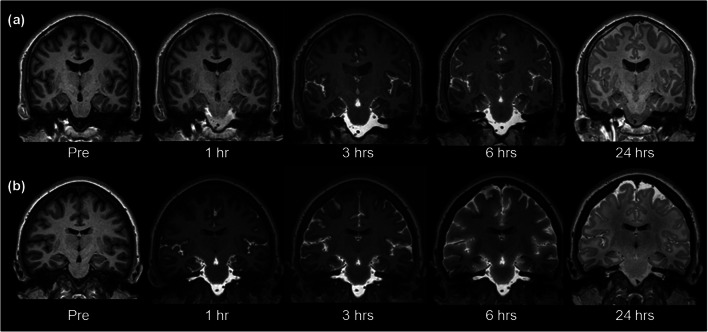
Intrathecal injection of gadobutrol in a dose of (**a**) 0.25 mmol (0.25 ml of 1.0 mmol/ml) or (**b**) 0.5 mmol (0.50 ml of 1.0 mmol/ml) at the lower lumbar level. Entry of gadobutrol in CSF within the intracranial compartment was preceded by unenhanced T1-weighted MRI (time point “Pre”), and acquisition of identical consecutive T1 scans was initiated immediately and repeated at different time points. Image window/level is adjusted to optimize qualitative illustration of CSF contrast enhancement

**Fig. 2:**
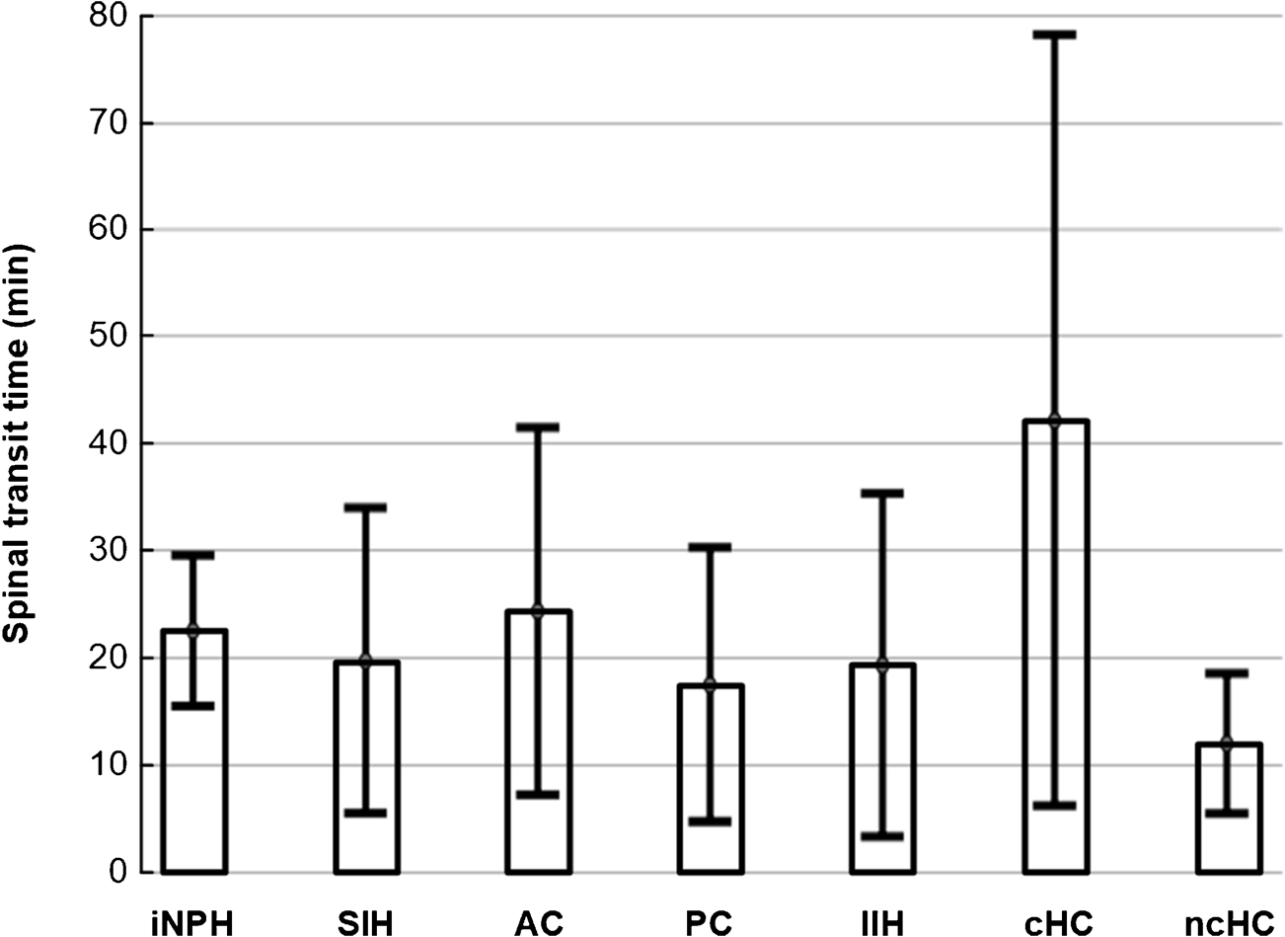
There was no significant difference between patient groups regarding time from intrathecal administration of gadobutrol until first enhancement of the contrast agent within the subarachnoid space of the foramen magnum (spinal transit time). For the entire group of 149 individuals, the mean ± stdev spinal transit time was 22 ± 34 min. The tentative diagnoses were as follows: iNPH, idiopathic normal pressure hydrocephalus; SIH, spontaneous intracranial hypotension; AC, arachnoid cyst; PC, pineal cyst; IIH, idiopathic intracranial hypertension; cHC, communicating hydrocephalus; and ncHC, non-communicating hydrocephalus

**Fig. 3:**
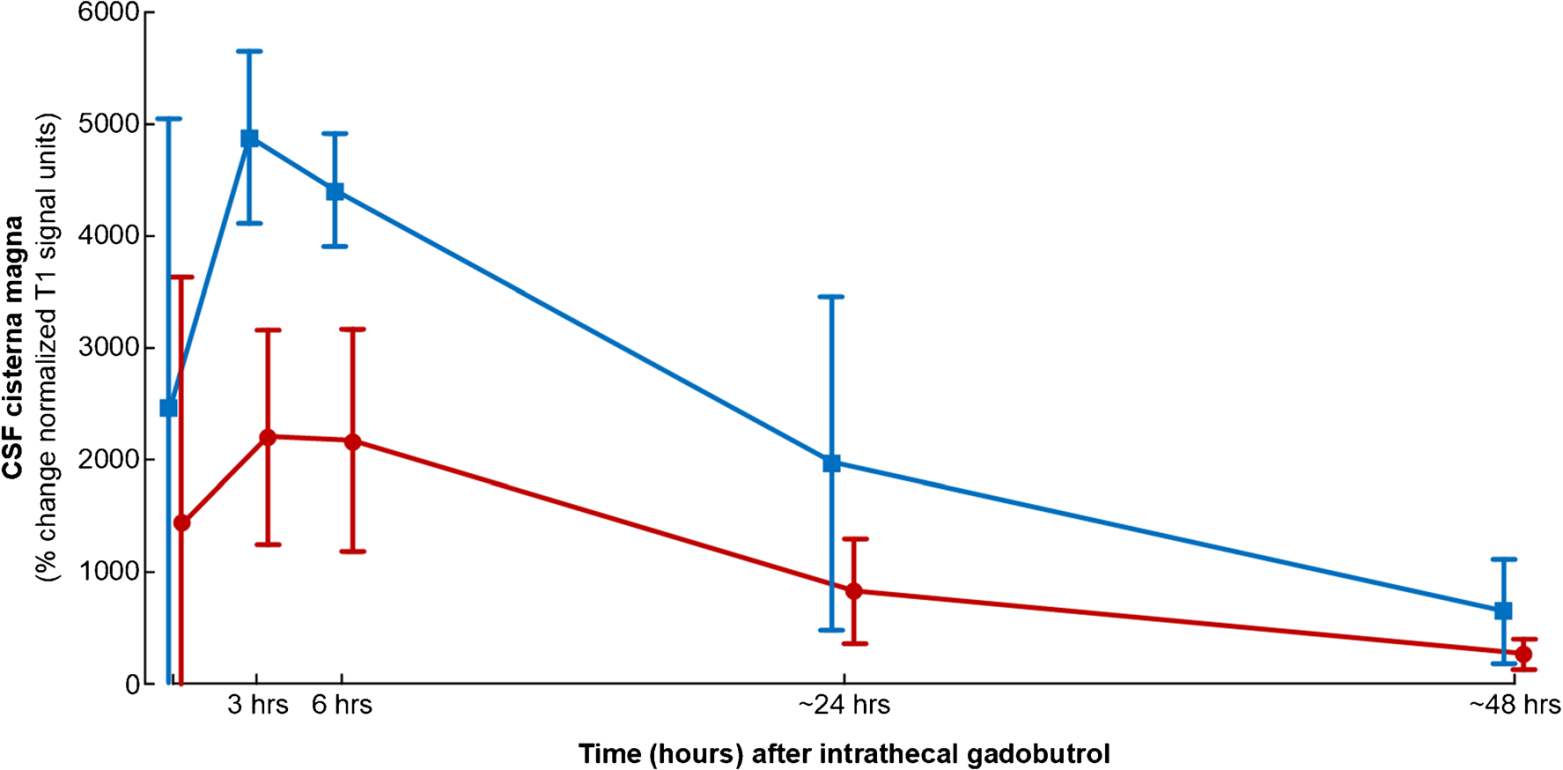
CSF tracer enrichment in cisterna magna depending on dose of CSF tracer (gadobutrol), measured as percentage change in normalized T1 signals after intrathecal injection of gadobutrol in doses of 0.5 mmol (protocols #1–2; blue line) or 0.25 mmol (protocol #3; red line). The first time point is after 10 min. The CSF tracer level was significantly different after 3 h (*P* < 0.001), 6 h (*P* = 0.001), and 48 h (*P* = 0.047; independent samples *t* test).

Entry of gadobutrol to cranial CSF spaces was verified in all individuals at MRI (Fig. 1). The time from intrathecal gadobutrol administration until first visual detection of the contrast agent in subarachnoid space at the level of foramen magnum was 22 ± 34 min (spinal transit time; Fig. 2), with no significant differences across the patient groups (Fig. 3).

The CSF enrichment within cisterna magna was dose-dependent with larger T1 intensity increase in CSF after 3, 6, and 48 h in individuals receiving intrathecal gadobutrol in a dose of 0.5 mmol as compared with 0.25 mmol (Fig. 3).

### Serious adverse events

Serious adverse events likely related to intrathecal gadobutrol administration were not seen in patients without a history of prior adverse events. By error, one woman with known allergy to iodinated contrast agents was included in the study and given the combination of intrathecal gadobutrol and iodixanol, despite interaction with referring doctor, study nurse, the neurologist who managed hospital admittance, and an anesthesiologist. She had an immediate anaphylactic reaction consisting of skin rash, dyspnea, and fall in blood pressure similar to the previous reaction to iodinated contrast agent. After intravenous Ringer acetate (1000 ml) and surveillance within the intensive care unit for a few hours, no further actions were needed, and she was able to undergo MRI scanning after a few hours.

An 80-year-old man with idiopathic normal pressure hydrocephalus (iNPH) was diagnosed with a pulmonary embolism, which was attributed to a long train journey and reduced mobility immediately before hospitalization.

### Non-serious adverse events depending on administration protocol

Table 2 lists the non-serious adverse events during days 1–3. Typically, these adverse events occurred during day 1 and rarely on days 2 or 3.

**Table 2:** Non-serious adverse events depending on protocol days 1–3

		Patients	Protocol				Statistics	
		Total (*n* = 149)	#1 (*n* = 115)	#2 (*n* = 29)	#3 (*n* = 5)	Protocol #1 vs. #2	Protocol #1 vs. #3	Protocol #2 vs. #3
No adverse events (*N*/%)		36 (24%)	26 (23%)	9 (31%)	1 (20%)	ns	ns	ns
Headache (*N*/%)		69 (46%)	52 (45%)	14 (48%)	3 (60%)	ns	ns	ns
Severity	Mild (*N*/%)	8 (5%)	4 (4%)	4 (14%)	0			
	Moderate (*N*/%)	17 (11%)	14 (12%)	2 (7%)	1 (20%)	ns	ns	ns
	Severe (*N/*%)	44 (30%)	34 (30%)	8 (28%)	2 (40%)			
Nausea (*N*/%)		84 (56%)	67 (58%)	13 (45%)	4 (80%)	ns	ns	ns
Severity	Mild (*N*/%)	25 (17%)	21 (18%)	1 (3%)	3 (60%)			
	Moderate (*N*/%)	11 (7%)	7 (6%)	4 (14%)	0	ns	ns	*P* = 0.004
	Severe (*N*/%)	48 (32%)	39 (34%)	8 (28%)	1 (20%)			
Dizziness (*N*/%)		60 (40%)	44 (38%)	13 (45%)	3 (60%)	ns	ns	ns
Severity	Mild (*N*/%)	20 (13%)	13 (11%)	5 (17%)	2 (40%)			
	Moderate (*N*/%)	16 (11%)	12 (10%)	4 (14%)	0	ns	ns	ns
	Severe (*N*/%)	24 (16%)	19 (17%)	4 (14%)	1 (20%)			
Itch/paresthesia (*N*/%)		20 (13%)	18 (16%)	2 (7%)	0	ns	ns	ns
Urinary incontinence (*N*/%)		1 (1%)	1 (1%)	0	0	-	-	-
Fatigue (*N*/%)		4 (3%)	1 (1%)	2 (7%)	1 (20%)	-	-	-
Vision problems (*N*/%)		2 (1%)	1 (1%)	1 (3%)	0	-	-	-
Cognitive impairment (*N*/%)		0	0	0	0	-	-	-
Back pain from spinal puncture (*N*/%*)*		18 (12%)	15 (13%)	2 (7%)	1 (20%)	ns	ns	ns
Muscular spasms (*N*/%)		1 (1%)	1 (1%)	0	0	-	-	-
Tremor (*N*/%)		1 (1%)	1 (1%)	0	0	-	-	-
Chills/warm feeling (*N*/%)		14 (9%)	11 (10%)	2 (7%)	1 (20%)	ns	ns	ns
Changed tasting experience (*N*/%)		3 (2%)	2 (2%)	1 (3%)	0	-	-	-

Non-serious adverse events were common, independent of administration protocol (Table 2). No symptoms at all after intrathecal contrast administration were reported by 23%, 31%, and 20% of patients for protocols #1, #2, and #3, respectively. The severity of nausea was weaker in individuals with protocol #3 as compared with protocol #2 (*P* = 0.004), providing preliminary evidence that occurrence of non-serious adverse events of intrathecal gadobutrol are dose-dependent. Otherwise, the occurrence of other non-serious adverse events did not differ significantly between protocols.

The occurrence of non-serious adverse events after 4 weeks and 12 months is presented in Table 3. After 12 months, two patients (1 %) reported urinary incontinence, but with unclear association to the administration protocol, as both had other diseases that might explain these complaints. No other individuals reported complaints related to participation in the project.

**Table 3:** Non-serious adverse events after 4 weeks and 12 months

		4 weeks	12 months
Headache (*N*/%)		10 (7%)	2 (1%)
Severity	Mild (*N*/%)	2 (1%)	0
	Moderate (*N*/%)	4 (3%)	0
	Severe (*N*/%)	4 (3%)	0
Nausea (*N*/%)		4 (3%)	0
Severity	Mild (*N*/%)	0	0
	Moderate (*N*/%)	1 (1%)	0
	Severe (*N*/%)	3 (2%)	0
Dizziness (*N*/%)		4 (3%)	0
Severity	Mild (*N*/%)	1 (1%)	0
	Moderate (*N*/%)	2 (1%)	0
	Severe (*N*/%)	1 (1%)	0
Itch/paresthesia (N/%)		4 (3%)	0
Urinary incontinence		2 (1%)	2 (1%)
Fatigue		1 (1%)	0
Vision problems		1 (1%)	0
Cognitive impairment		0	0
Back pain from spinal puncture		2 (1%)	0
Muscular spasms		0	0
Tremor		0	0
Chills/warm feeling		0	0
Changed tasting experience		0	0

Data presented as number of individuals (percentage in parenthesis)

### Non-serious adverse events depending on clinical indication for MRI

The non-serious adverse events during days 1–3 heavily depended on clinical diagnosis (Table 4). While 37% of individual with iNPH did not reported any non-serious adverse events, the same numbers were 0% and 14% for individuals with a pineal gland cyst or idiopathic intracranial hypertension (IIH), respectively. The occurrence and severity of headache, nausea, and dizziness differed significantly between clinical diagnoses (Table 4).

**Table 4:** Non-serious adverse events days 1–3 depending on clinical indication for intrathecal gadobutrol

		Days 1–3							
		iNPH (*n* = 38)	SIH (*n* = 22)	AC (*n* = 27)	PC (*n* = 26)	IIH (*n* = 22)	cHC (*n* = 11)	ncHC (*n* = 3)	Significance
No non-serious adverse events (*N*/%)		14 (37%)	5 (23%)	10 (37%)	0	3 (14%)	3 (27%)	1 (33%)	*P* = 0.016
Headache (*N*/%)		6 (16%)	10 (46%)	9 (33%)	22 (85%)	15 (68%)	6 (55%)	1 (33%)	*P* < 0.001
Severity	Mild (*N*/%)	1 (3%)	2 (9%)	0	0	5 (23%)	0	0	
	Moderate (*N*/%)	3 (8%)	5 (23%)	3 (11%)	4 (15%)	0	2 (18%)	0	*P* < 0.001
	Severe (*N*/%)	2 (5%)	3 (14%)	6 (22%)	18 (69%)	10 (46%)	4 (36%)	1 (33%)	
Nausea (*N*/%)		18 (47%)	11 (50%)	12 (44%)	21 (81%)	14 (64%)	6 (55%)	2 (67%)	ns
Severity	Mild (*N*/%)	7 (18%)	6 (27%)	5 (19%)	3 (12%)	1 (5%)	2 (18%)	1 (33%)	
	Moderate (*N*/%)	1 (3%)	2 (9%)	2 (7%)	1 (4%)	4 (18%)	1 (9%)	0	*P* = 0.044
	Severe (*N*/%)	10 (26%)	3 (14%)	5 (19%)	17 (65%)	9 (41%)	3 (27%)	1 (33%)	
Dizziness (*N*/%)		6 (16%)	7 (32%)	12 (44%)	20 (77%)	12 (55%)	2 (18%)	1 (33%)	*P* < 0.001
Severity	Mild (*N*/%)	5 (13%)	4 (18%)	5 (19%)	3 (12%)	3 (14%)	0	0	
	Moderate (*N*/%)	1 (3%)	2 (9%)	2 (7%)	7 (27%)	3 (14%)	0	1 (33%)	*P* < 0.001
	Severe (*N*/%)	0	1 (5%)	5 (19%)	10 (39%)	6 (27%)	2 (18%)	0	
Itch/paresthesia (*N*/%)		2 (5%)	4 (18%)	3 (11%)	8 (31%)	1 (5%)	1 (9%)	1 (33%)	ns
Urinary incontinence		0	1 (5%)	0	0	0	0	0	
Fatigue		0	0	1 (4%)	2 (8%)	1 (5%)	0	0	ns
Vision problems		0	0	0	0	1 (5%)	1 (9%)	0	
Cognitive impairment		0	0	0	0	0	0	0	
Back pain from spinal puncture		1 (3%)	1 (5%)	3 (11%)	8 (31%)	3 (14%)	2 (18%)	0	*P* = 0.032
Muscular spasms		0	1 (5%)	0	0	0	0	0	
Tremor		0	1 (5%)	0	0	0	0	0	
Chills/warm feeling		3 (8%)	1 (5%)	2 (7%)	5 (19%)	3 (14%)	0	0	ns
Changed tasting experience		0	0	0	1 (4%)	1 (5%)	0	1 (33%)	

Data presented as number of individuals (percentage in parenthesis). Significance determined by the Pearson chi-square test. Data presented as number of individuals (percentage in parenthesis). *iNPH* idiopathic normal pressure hydrocephalus, *SIH* spontaneous intracranial hypotension, *AC* arachnoid cyst, *PC* pineal cyst, *IIH* idiopathic intracranial hypertension, *cHC* communicating hydrocephalus, *ns* non-significant

After 4 weeks, particularly the occurrence and severity of headache differed significantly between diagnosis categories (Table 5). Among individuals with pineal cysts and IIH, 12% and 18% reported headache.

**Table 5:** Non-serious adverse events after 4 weeks depending on clinical indication for intrathecal gadobutrol

		4 weeks							
		iNPH (*n* = 38)	SIH (*n* = 22)	AC (*n* = 27)	PC (*n* = 26)	IIH (*n* = 22)	cHC (*n* = 11)	ncHC (*n* = 3)	Significance
No non-serious adverse events (*N*/%)		35 (92%)	19 (86%)	24 (89%)	21 (81%)	17 (77%)	10 (91%)	2 (67%)	ns
Headache (*N*/%)		0	1 (5%)	1 (4%)	3 (12%)	4 (18%)	0	1 (33%)	*P* = 0.042
Severity	Mild (*N*/%)	0	0	1 (4%)	0	0	0	1 (33%)	
	Moderate (*N*/%)	0	1 (5%)	0	1 (4%)	2 (9%)	0	0	*P* = 0.002
	Severe (*N*/%)	0	0	0	2 (8%)	2 (9%)	0	0	
Nausea (*N*/%)		0	0	0	1 (4%)	3 (14%)	0	0	
Severity	Mild (*N*/%)	0	0	0	0	0	0	0	
	Moderate (*N*/%)	0	0	0	0	1 (5%)	0	0	ns
	Severe (*N*/%)	0	0	0	1 (4%)	2 (9%)	0	0	
Dizziness (*N*/%)		1 (3%)	0	1 (4%)	0	2 (9%)	0	0	
Severity	Mild (*N*/%)	0	0	0	0	1 (5%)	0	0	
	Moderate (*N*/%)	1 (3%)	0	1 (4%)	0	0	0	0	ns
	Severe (*N*/%)	0	0	0	0	1 (5%)	0	0	
Itch/paresthesia (*N*/%)		1 (3%)	1 (5%)	1 (4%)	0	1 (5%)	0	0	
Urinary incontinence		0	1 (5%)	0	1 (4%)	0	0	0	
Fatigue		1 (3%)	0	0	0	0	0	0	
Vision problems		0	0	0	0	1 (5%)	0	0	
Cognitive impairment		0	0	0	0	0	0	0	
Back pain from spinal puncture		0	0	1 (4%)	1 (4%)	0	0	0	
Muscular spasms		0	0	0	0	0	0	0	
Tremor		0	0	0	0	0	0	0	
Chills/warm feeling		0	0	0	0	0	0	0	
Changed tasting experience		0	0	0	0	0	0	0	

Data presented as number of individuals (percentage in parenthesis). Significance determined by the Pearson chi-square test. Data presented as number of individuals (percentage in parenthesis). *iNPH* idiopathic normal pressure hydrocephalus, *SIH* spontaneous intracranial hypotension, *AC* arachnoid cyst, *PC* pineal cyst, *IIH* idiopathic intracranial hypertension, *cHC* communicating hydrocephalus, *ns* non-significant

## Discussion

The main result of this prospective study is that intrathecal gadobutrol in doses of either 0.25 or 0.5 mmol caused no serious adverse events in individuals without prior history of allergic reactions to contrast agents. The frequency of non-serious adverse events after days 1–3 or 4 weeks was primarily determined by the tentative patient diagnosis prior to intrathecal injection. There were no definitive adverse events after 12 months.

The clinical indications for intrathecal gadobutrol in this study were various forms of suspected CSF circulation disorders, where gadobutrol at the same time served as a CSF tracer as part of our gMRI protocol [21]. We administered gadobutrol off-label based on special permission from the National Medicines Agency of Norway, in a standardized research setting only. However, intrathecal MRI contrast agents in similar concentrations have previously been used clinically off-label to visualize CSF leakage in individuals with spontaneous intracranial hypotension (SIH) [22, 23] and otorhinorrhea [24], as well as in the diagnostic assessment of disorders such as arachnoid cysts [25] and iNPH [26].

The safety profile of gadolinium-based contrast agents was recently reviewed in this journal; the authors concluded that no cause-effect relationship has been shown in humans regarding brain gadolinium exposure and clinical consequences specific to neurological toxicity [8]. Previously, several studies found the linear contrast agent gadopentetic acid to have low risk when given intrathecally to patients in a dose 0.5–1.0 mmol [22, 27–31]. A recent systematic review concluded that serious adverse events of intrathecal administered MRI contrast agents are highly dose-dependent; the observed serious adverse events were seen after administration of 2 mmol or higher doses [10]. Non-serious adverse events, primarily headache, occurred following doses of 1 mmol or lower, but they were not able to conclude about the cause since headache occurs post lumbar puncture in about 49% of patients [10]. Headache also was the most common non-serious adverse event in the present study. In comparison, intrathecal gadopentetic acid caused headache in 10/36 (27%) iNPH patients [26] and in 6/20 (30%) patients with arachnoid cysts [31].

The available evidence points to a small gap between advantageous diagnostic effects and neurotoxic effects of intrathecal MRI contrast agents. From our experience, intrathecal gadobutrol in a dose of 0.5 mmol or less is safe, which in an adult with a brain weighing 1400 g represents an exposure 0.36 μmol/g gadobutrol to brain. Intrathecal gadobutrol in a dose of 2.0 mmol or higher may cause neurotoxic effects. It was previously reported in humans that overdose of gadopentetic acid (6–20 times normal dose) could be neurotoxic [32, 33]. One individual who by accident received 20 mmol gadopentetic acid (7.0 μmol/g brain) developed neurological deficits (speech problems, visual impairment, fatigue, and psychotic symptoms) lasting 2 weeks, but symptoms had disappeared after 2 months [34]. Intrathecal gadobutrol in a dose of 2.0 mmol caused spastic pain of the lower extremities [35]. Recently, another study reported fatal outcome in a 67-year-old person who by accident received 2.0 mmol gadoteridol, corresponding to a concentration 2.3 μmol/g brain [36]. The authors reviewed the literature about neurotoxic effects of intrathecal gadolinium; the literature reports no neurological complications after doses causing concentration of < 1.0 μmol/g brain [36].

Taken together, the existing data suggest intrathecal gadobutrol in a dose of 0.5 mmol (corresponding to a dose of 0.36 μmol gadobutrol per gram brain) to be safe. Nevertheless, the present study provides preliminary data that gadobutrol in a dose of 0.25 mmol causes less severe nausea than 0.5 mmol. Since only five individuals received 0.25 mmol, this need to be further studied. We further note that intrathecal gadobutrol in a dose of 0.25 mmol provided strong enrichment within CSF of cisterna magna, though the CSF tracer level was significantly lower than 3, 6, and 48 h after a dose of 0.5 mmol. Nevertheless, a dose of 0.25 mmol may be considered sufficient for diagnosis of CSF leaks from the spinal canal or at the skull base. We will explore the clinical utility of this dose in future studies where intrathecal gadobutrol is used as CSF tracer to assess the glymphatic system and craniospinal molecular clearance capacity.

It should be noted that the present comparison of different administration protocols was hampered by the diagnostic categories allocated to the respective administration protocols. The frequency of non-serious adverse events heavily depended on the clinical indication for intrathecal gadobutrol. It is well recognized that IIH and symptomatic pineal gland cysts may sometimes be accompanied with severe symptoms such as headache, nausea, and dizziness [37, 38], which overlap with symptoms typically seen after intrathecal contrast agent injections. Furthermore, headache is a predominant symptom in individuals with symptomatic arachnoid cysts [39]. Adding iohexanol as in protocol #1 would be expected to increase occurrence of adverse events [40]. This effect was, however, not seen here, probably related to the substantial contribution of symptoms from the diagnoses pineal gland cyst, IIH, and arachnoid cyst to the adverse event profile. Taken together, given the high proportion of individuals with a pineal cyst or IIH in protocol #2 (42%) than in protocol #1 (30%; Table 1), this probably explains the higher frequency of the non-serious adverse events headache, nausea, and dizziness in days 1–3 with protocol #2.

One concern regarding intrathecal use of MRI contrast agents is the potential for deposition within brain parenchyma. In a previous study, 4 week after one single intrathecal administration of 0.5 mmol gadobutrol, we found no detectable change in normalized T1 signal in any brain region, including basal ganglia indicative of no retention [21]. Moreover, it is important to bear in mind that it is now established that after an ordinary clinical intravenous dose of gadolinium, the contrast agent is present in CSF samples for days and weeks, even despite normal renal function and intact blood-brain barrier [14, 15]. One study of rodents reported that the concentration of MRI contrast agent in CSF exceeded that of blood 4.5 h after intravenous administration [17]. A typical intravenous dose (0.1 mmol/kg in an 80-kg patient = 8 mmol and with half-time in blood being approximately 2 h) may therefore, theoretically, render for a notable passage of contrast agent to CSF, as recently shown in an MRI study [16]. The difference in peak CSF concentration of gadobutrol after conventional intravenous dose compared with intrathecal doses of 0.5 or 0.25 mmol remains to be determined. This may potentially be quantified with MRI using T1-maps, allowing for quantification of gadobutrol concentration in different locations intracranially.

The clinical use of intrathecal MRI contrast agents would require acceptable balance between safety and added diagnostic value, as compared with best available methods. Given the probable small therapeutic index of intrathecal MRI contrast agents, the relative safety and diagnostic advantage need to be further studied.

## Conclusion

This prospective study showed that intrathecal administration of 0.5 and 0.25 mmol gadobutrol was safe. Non-serious adverse events were only to a small degree affected by different administration protocols. Preliminary data are given that a lower dose 0.25 mmol of intrathecal gadobutrol may be favorable, but this needs to be further studied. Frequency of non-serious adverse events was highest among patients under work-up for IIH and pineal gland cysts, who typically present with symptoms comparable with some of those reported after intrathecal administration of contrast agents.
